# Preventive effect of ferulic acid on dextran sulfate sodium-induced ulcerative colitis in mice

**DOI:** 10.3389/fmicb.2026.1767221

**Published:** 2026-04-13

**Authors:** Zheng Zhong, Zhihan Dong, Hongya Zheng, Yanzhu Zhu, Caoxing Huang, Wei Lian, Baishuang Yin

**Affiliations:** 1 College of Animal Science and Technology, Heilongjiang Bayi Agricultural University, Daqing, China; 2 Key Lab of Preventive Veterinary Medicine in Jilin Province, College of Animal Science and Technology, Jilin Agricultural Science and Technology University, Jilin, China; 3 Key Laboratory of Medicinal Materials, Jilin Academy of Chinese Medicine Sciences, Changchun, China; 4 College of Chemical Engineering, Nanjing Forestry University, Nanjing, China; 5 Jilin Zhengye Biological Products Ltd. Co., Jilin, China

**Keywords:** antioxidant capacity, ferulic acid, gut microbiota, mice, ulcerative colitis

## Abstract

**Introduction:**

Ulcerative colitis (UC) is one of the predominant forms of inflammatory bowel disease (IBD), and it is a chronic and recurring inflammation of the gastrointestinal trac. However, the therapies are associated with drug resistance and low responsiveness. Ferulic acid (FA) alleviates clinical signs and inflammatory mediators in UC, while the molecular mechanisms of FA regulation on UC remain elusive. The aim of this experiment is to ascertain the preventive mechanism of FA on the non-infectious UC.

**Methods:**

Sixty male Kunming mice (6–8 weeks) were randomly allocated into six groups: Control (CON), Dextran sulfate sodium (DSS), FA+DSS, FA, Prednisone (Pdn), and Pdn+DSS group. Colonic histopathology, Interleukin-1β (IL-1β), interleukin-6 (IL-6), IL-10, IL-2 concentrations, fecal calprotectin (FC), fecal lactoferrin (FL), superoxide dismutase (SOD), glutathione peroxidase (GSH-Px), catalase (CAT) activities, MDA content and total antioxidant capacity (T-AOC) were quantified by HE staining, transmission electron microscopy, and ELISA Kit. Gut microbiota composition was analyzed by sequencing of the 16S rRNA V4 gene region.

**Results:**

Compared with the DSS group, FA administration significantly reduced IL-1β and IL-6 levels, FC and FL, promoted the increase of IL-2 and IL-10, markedly elevated activities of SOD, GSH-Px, CAT and total antioxidant capacity (T-AOC) activity, and decreased MDA levels. Moreover, preventive FA supplementation improved extensive inflammation infiltration and crypt destruction in colon of UC mice, mitochondrial damage in the colonic epithelial cells. Preventive FA supplementation exhibited a return to original levels of Muribaculaceae and Clostridia.

**Conclusion:**

Collectively, FA and Pdn effectively attenuated DSS-induced UC by restoring colonic morphology, suppressing inflammatory mediators, enhancing antioxidant capacity, and regulating gut microbiota. These findings provide a promising strategy for the expanded application of FA in UC prevention.

## 1 Introduction

Ulcerative colitis (UC) is a chronic, lifelong, non-specific intestinal inflammatory disease, which is charactered with abdominal pain, chronic diarrhea, and rectal bleeding. The incidence of UC in emerging industrialized countries and regions such as Asia, South America and Eastern Europe has shown a rapid growth trend (Ashique et al., 2023; Yamazaki et al., 2023). While the precise etiology of UC remains elusive, the most accepted pathogenesis involves chronic intestinal inflammation. Intestinal microbiota played an important role in the pathogenesis and progression of UC, including decreased microbial diversity, reduced beneficial bacteria and increased harmful bacteria (Wu et al., 2023). While current anti-inflammatory therapies aim to control symptoms, many patients experience drug resistance and low responsiveness. Prednisone (Pdn) is a synthetic glucocorticoid and one of the key medications for treating UC. Although inflammation symptoms could be alleviated by glucocorticoids, metabolic disorders, endocrine abnormalities, hepatic injury, and severe pancreatitis were often observed (Di Rienzo et al., 2024). It is necessary to explore safe and effective natural active ingredients for UC as potential intervention strategies.

Ferula is a resin derived from the plants Ferula Xinjiang or Ferula fukang of the Apiaceae family (Hu et al., 2024). Historically, Ferulic acid (FA) was first isolated and identified from the components of such plants, and thus it got its name (Martínková et al., 2023). It exhibited anti-inflammatory, antioxidant, anticancer, and antibacterial effects (Zhang et al., 2022; Zhang et al., 2023). FA enhanced autophagy, inhibited the activation of NLRP3 inflammasome and reduced the expression and release of inflammatory factors (Liu et al., 2022). FA had a gastroprotective effect against indomethacin-induced gastric ulcers in rats due to its antioxidant and anti-inflammatory properties (Ermis et al., 2023). FA inhibits the inflammatory injury of endothelial cells in 2,4,6-triabrobenzene sulfonic acid-induced UC (Yu S. et al., 2023). However, its role in the other UC model is still unclear.

In this study, the non-infectious UC in mice was established by dextran sulfate sodium (DSS). The protective effect of FA on DSS-induced UC was explored through colonic morphology, inflammatory mediators, antioxidant capacity, and gut microbiota compared with the Pdn. It will provide a theoretical basis for the expanded application of FA in UC prevention.

## 2 Materials and methods

### 2.1 Reagents

The molecular weight of DSS is 36–50 kda and it was purchased from Yeasen Biotechnology (Shanghai) Co., Ltd. Item No. 60316ES76. Pdn was purchased from Shanghai Maokang Biotechnology Co., Ltd. Item number: MZ10301-25G. Qinglong PI is extracted from the green husks of walnuts in Changbai Mountain (jilin) by Nanjing Forestry University.

### 2.2 Experimental animal

**Figure 1:**
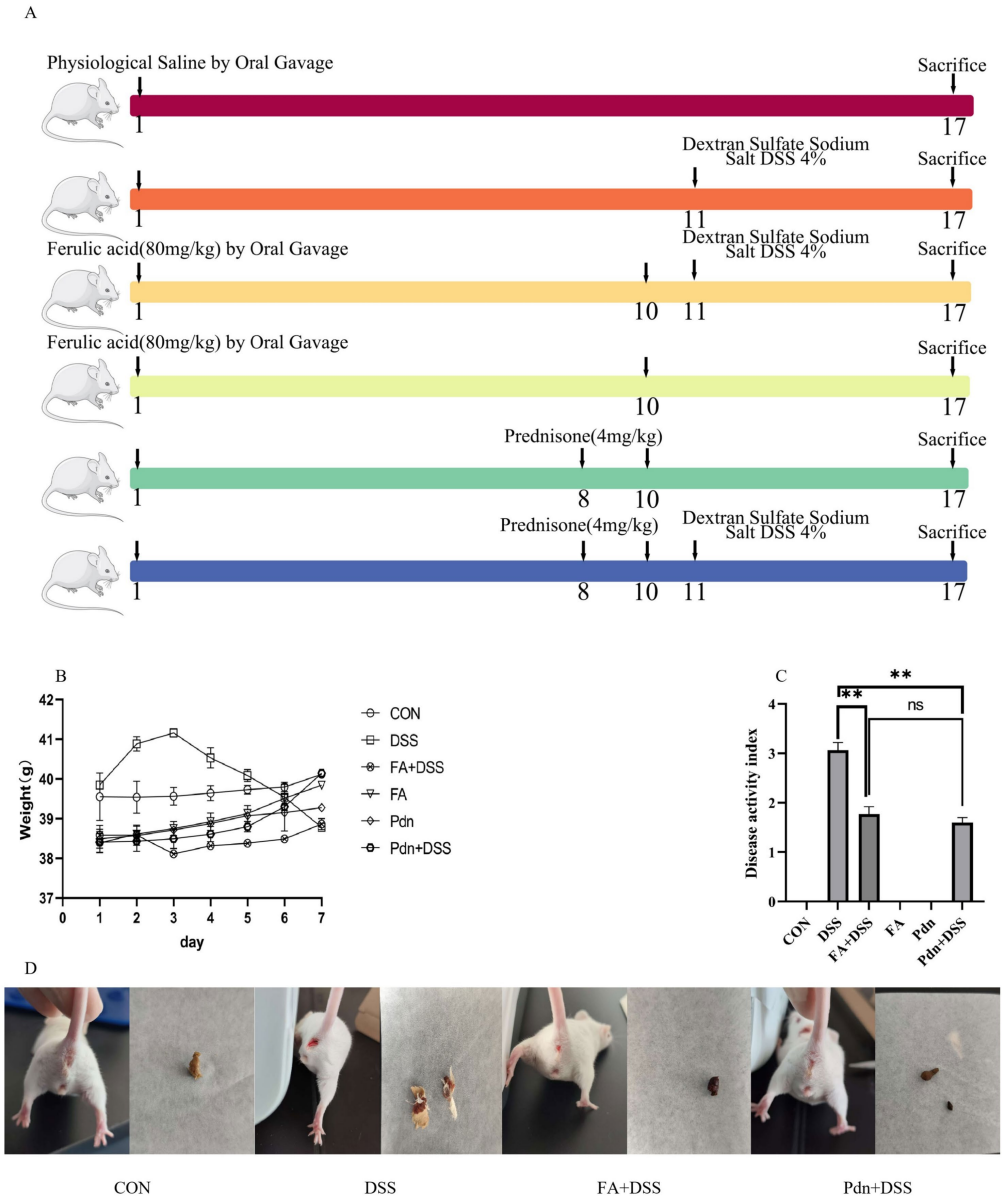
The clinical symptom evaluation (*n* **=** 10). **(A)** Experimental flowchart, **(B)** body weight of mice in each group was monitored daily for 7 days, **(C)** DAI disease score was calculated, **(D)** the main symptoms of UC were captured.

Sixty male Kunming mice (6–8 weeks old and weighing 20 ± 2 g) were housed in ventilated cages under controlled conditions (23 ± 2 °C; 50–60% humidity; 12-h light/dark cycle). The standard food and water were available *ad libitum,* and bedding were replaced every other day. After a 7-day acclimatization, the mice were randomly allocated to six groups (*n* = 10, oral gavage): Control group (CON), Dextran sulfate sodium group (DSS, 3%), FA group (80 mg/kg), FA+DSS group, Pdn (4 mg/kg), Pdn+DSS group (Figure 1A). The diagram illustrated the experimental flowchart of this study (Figure 1A). DSS (3%) gavage was freshly prepared daily to the mice in the DSS, FA+DSS, and Pdn+DSS groups for 7 days. On the third day before DSS exposure, FA (extracted from Changbai Mountain Qinglongyi) was administered by gavage once daily for 10 days. On the fourth day before DSS exposure, Pdn was given by gavage once daily for 3 days. Body weight was recorded daily during experiment. At the end of the experiment, mice were euthanized under isoflurane anesthesia. Whole blood was collected from the retro-orbital plexus and centrifuged at a speed of 1, 400 × g for 10 min to obtain serum. The entire colon was excised, and gently rinsed with ice-cold normal saline to remove luminal contents without damaging the mucosa. Colon segments (2 cm) were immobilized in a 4% formaldehyde solution. Colon samples (1 mm^3^) were immediately immersed in ice-cold 2.5% glutaraldehyd. The dose of DSS was chosen according to Lu’s experiment (Lu et al., 2022). The doses of FA and Pdn was chosen according to Huang and Mencarelli’s experiment, respectively (Huang et al., 2025; Mencarelli et al., 2016).

### 2.3 Detection of disease activity index (DAI)

The Disease Activity Index (DAI) scores in the mice were calculated according to DAI method (Zhang et al., 2024). The Disease Activity Index (DAI) scores were observed as follows: (a) weight loss: 0 points = not available, 1 point = 1–5%, 2 points = 5–10%, 3 points = 10–15%, and 4 points = more than 15%; (b) fecal consistency: 0 point = normal, 1 point = not rigorous, 2 points = soft stool, 3 points = meager stool, and 4 points = constipation; (c) hemorrhage: 0 points = normal, 1 point = negative fecal occult blood count, 2 points = positive for occult blood, 3 points = obvious bloody stool, and 4 points = profuse bleeding.

### 2.4 Histopathological examination of mouse colon

Colon segments (2 cm) were immobilized in a 4% formaldehyde solution. A standard ethanol–xylene gradient was used for dehydration and clearing before paraffin infiltration. Fixed colon tissue was embedded in Paraffin. Serial 5 μm sections were cut on a rotary microtome, mounted on glass slides, and stained with hematoxylin and eosin method. Slides were covers with mounting medium and examined under a light microscope (Zhang et al., 2021). The histological scoring was evaluated by two independent blinded investigators.

### 2.5 The ultrastructural changes of colon tissue

Colon samples (1 mm^3^) were immediately immersed in ice-cold 2.5% glutaraldehyde for 2 h. The tissue was post-fixed with 1% osmium tetroxide for 2 h. Specimens were dehydrated through graded ethanol solutions, infiltrated with epoxy resin (Epon 812), and polymerized at 60 °C for 48 h. Ultrathin sections (70 nm) were cut on an ultramicrotome, mounted on copper grids, and contrast-stained with uranyl acetate followed by lead citrate. Ultrastructural changes in colon were examined with a transmission electron microscope (Zhu et al., 2016). And the mitochondria histological scoring was evaluated by two independent blinded investigators.

### 2.6 Detection of colitis factors in mice

Serum levels of IL-1β (YJ301814), IL-6 (YJ098430), IL-2 (YJ35348), and IL-10 (YJ34730) were quantified using ELISA kits (Shanghai Yuanju Biotechnology Center, Shanghai, China) according to the manufacturer’s instructions. Additionally, the levels of lactoferrin (FL) (ml821520) and fecal calprotectin (FC) (ml106769L) in mouse feces were measured using ELISA kits (Shanghai Enzyme-linked Biotechnology Co., Ltd., Shanghai, China).

### 2.7 Detection of antioxidant factors in mice

To evaluate the oxidative stress and antioxidant defense status of the experimental subjects, the enzymatic activities of superoxide dismutase (SOD, kit catalog no. A001-3-2), glutathione peroxidase (GSH-Px, kit catalog no. A005-1-2), catalase (CAT, kit catalog no. A007-1-1), and total antioxidant capacity (T-AOC, kit catalog no. A015-2-1), as well as the content of malondialdehyde (MDA, kit catalog no. A003-2-2) in the collected serum samples, were quantitatively determined using commercially available assay kits. All these kits were purchased from Nanjing Jiancheng Bioengineering Institute (Nanjing, Jiangsu Province, China), and the entire detection process was strictly performed in accordance with the detailed standard operating protocol.

### 2.8 Western blot analysis

The protein expression of NLRP3 was examined using western blot (Zhu et al., 2020). The supernatants of the colon were collected and determined using a BCA Protein Assay Kit (Beyotime Bio, Beijing, China). 40 μg of protein was separated on 10% sodium dodecyl sulfate–polyacrylamide gels, subjected to electrophoresis and transferred to PVDF membranes (polyvinylidene difluoride). The PVDF membranes were blocked with 5% non-fat milk for 2 h at room temperature, followed by incubation overnight with primary antibodies of NLRP3 (1:2000, 68,102-1-Ig, Proteintech Group, Inc., Wuhan, China) and *β*-actin (1:5000, 20,536-1-AP, Proteintech Group, Inc., Wuhan, China), on a shaker at 4 °C. Then, the membranes were washed three times with TBS-T and incubated with anti-rabbit horseradish peroxidase-conjugated IgG (1:5000, R00001, Nature Biosciences, Hangzhou, China) for 2 h at room temperature. Subsequently, PVDF were washed three times with TBS-T. The immunoreactivity was detected using an enhanced chemiluminescence reaction. The density of the bands was quantified by Image J version 2.0 (USA). β-actin was used as an internal control to normalize protein loading, and the relative expression of NLRP3 was calculated as the ratio of NLRP3 band density to β-actin band density.

### 2.9 Gut microbiota sequencing

Fresh luminal contents were aseptically collected from the rectum of each group immediately after euthanasia. Microbial genomic DNA was isolated using the QIAamp Fast DNA Stool Mini Kit (Qiagen, Hilden, Germany) according to the manufacturer’s protocol. Gut microbiota composixxtion was analyzed by sequencing of the 16S rRNA V4 gene region. The primers used were 515F (GTGCCAGCMGCCGCGGTAA) and 806R (GGACTACHVGGGTWTCTAAT). Sequencing was conducted on the NovaSeq 6,000 platform (Illumina) using 2 × 250-bp paired-end sequencing with the NovaSeq 6,000 SP Reagent Kit (Illumina) (500 cycles).

### 2.10 Statistical analysis

The results were analyzed by one-way analysis of variance followed by Tukey’s HSD (SPSS 20.0 software; SPSS Inc., Chicago, IL, USA). The histograms were drawn by the GraphPad Prism (version 8.0, GraphPad Software Inc., San Diego, CA, USA). The results were expressed as mean ± standard deviation (SD). SD was shown on the top of each column. ***p < 0.001 indicated extremely significant difference, **p < 0.01 indicated markedly significant difference, *p < 0.05 indicated significant difference, and *p* > 0.05 was considered not statistically significant.

## 3 Experimental results

### 3.1 Animal model analysis

The average body weight increased in the FA+DSS group (Figure 1B). Seven days after DSS induced exposure, diminished food and water intake, increased irritability, weight loss, and mucoid or bloody diarrhea were documented. The Disease Activity Index (DAI) is a standardized quantitative scoring system used to assess the severity of UC. Based on these symptoms, the DAI disease scores in the DSS modeling exhibited increased trend, while a reduction in DAI disease scores was observed in the FA and Pdn group (Figure 1C). Compared with the CON, the mice in the DSS group had obvious hematochezia conditions, while the hematochezia conditions of the mice in the FA and the Pdn prevention group were significantly alleviated (Figure 1D). As indicated by the above results, the DSS modeling was successful in this experiment.

### 3.2 Inflammatory cytokines in mice

**Figure 2:**
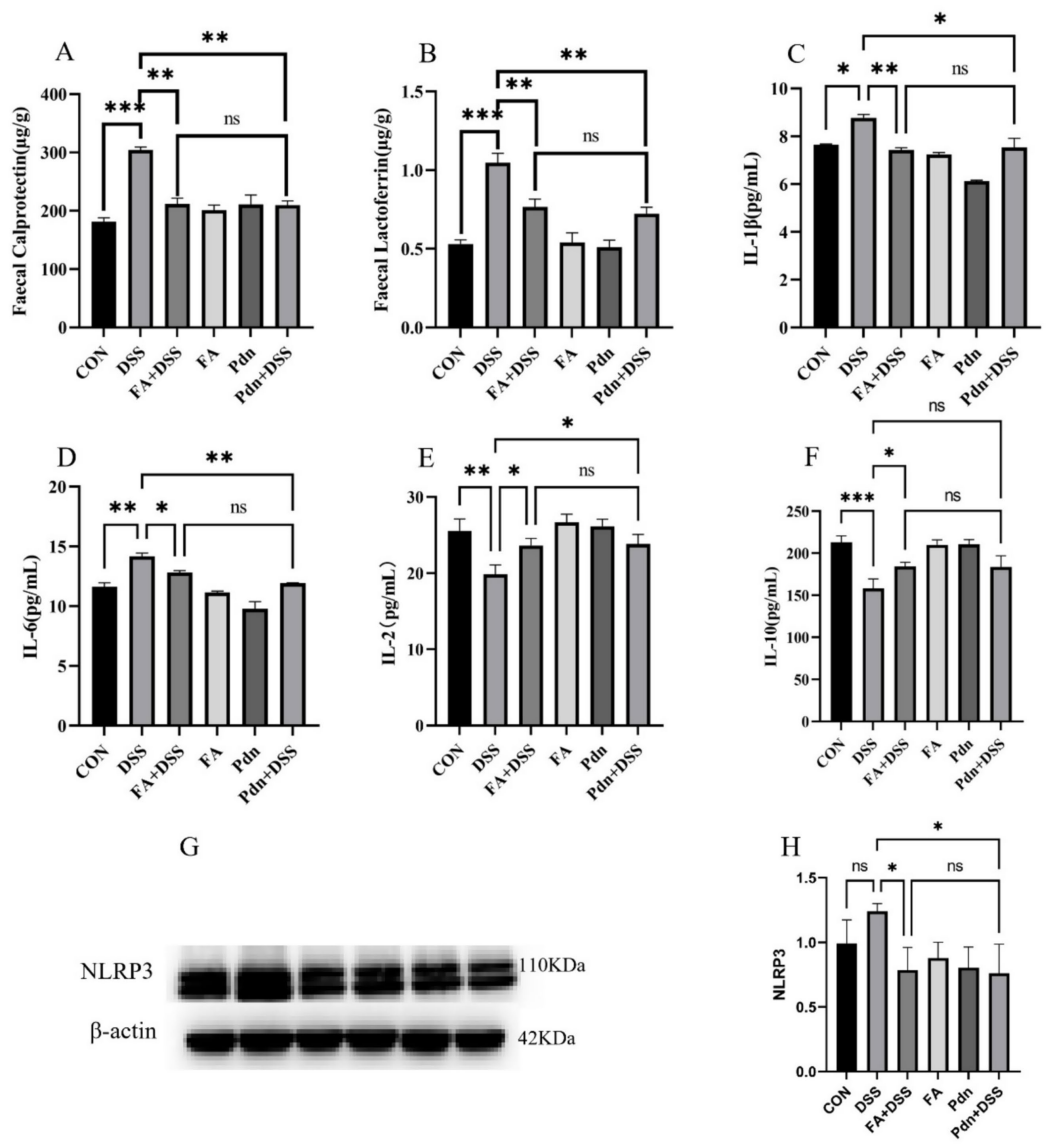
Preventive FA supplementation on the inflammation cytokine and anti- inflammation cytokine (*n* = 10 per group). FC, FL, IL-1β, IL-6, IL-2, and IL-10 were quantified using ELISA kits. **(A)** FC, **(B)** FL, **(C)** the IL-1β level, **(D)** the IL-6 level, **(E)** the IL-2 level, **(F)** the IL-10 level, **(G)** NLRP3 protein expression, and **(H)** mean gray value analysis of NLRP3 protein expression. All data were expressed as the mean ± SD. **p < 0.01, *p < 0.05, and ns *p* > 0.05. SD was shown on the top of each column.

FC and FL are standard non-invasive biomarkers for the clinical diagnosis of UC. IL-1β, IL-2, IL-6, and IL-10 plays the core roles of initiating inflammation balance. Compared with the DSS group, such increases of FC and FL were significantly suppressed by preventive supplementation with FA and Pdn (*p* < 0.01, Figures 2A,B). This increase of IL-6 and IL-1β was markedly reduced when FA and Pdn were preventively supplemented (*p* < 0.01, Figures 2C,D). The level of IL-2 and IL-10 was increased by preventive supplementation with both FA and Pdn (*p* < 0.05, Figures 2E,F). These results indicate that preventive supplementation of FA and Pdn can effectively alleviate inflammatory responses, effectively preventing UC.

### 3.3 NLRP3 protein expression in colon of mice

In UC, the NLRP3 inflammasome is a core inflammatory sensor and amplifier, and its excessive activation is a key link driving intestinal immune imbalance and tissue damage. As presented in Figures 2G,H, increase trend of the NLRP3 protein expression were reduced in the FA+DSS group and the Pdn+DSS group (*p* < 0.05). This suggests that preventive FA and supplementation can inhibit NLRP3 protein expression in the DSS group.

### 3.4 Antioxidant function in serum of mice

**Figure 3:**
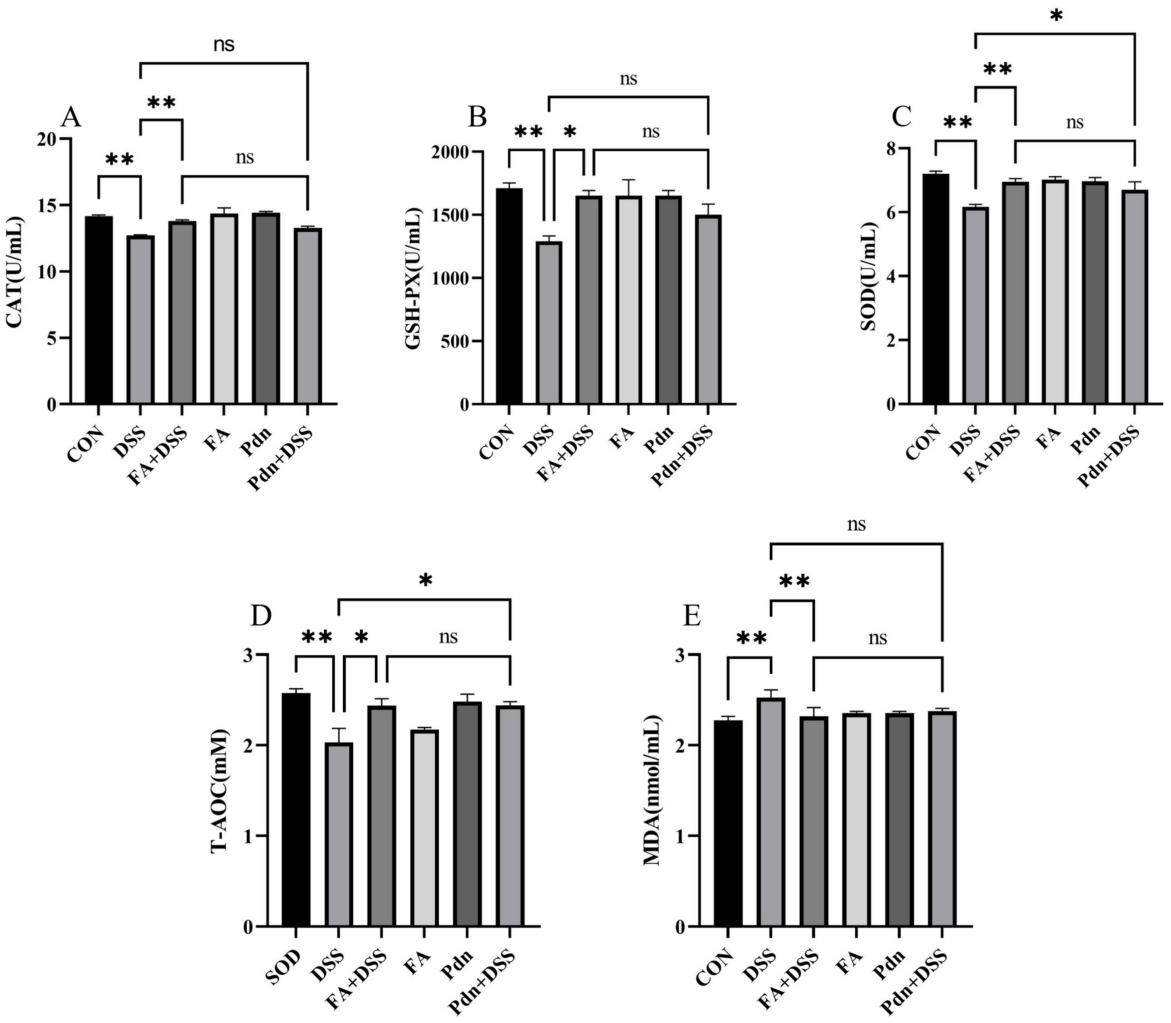
Preventive FA supplementation on the anti-oxidative function (*n* = 10 per group). The activity of SOD, GSH-Px, CAT, T-AOC activity, and MDA content were measured using Commercial kits. **(A)** The CAT activity, **(B)** the GSH-Px activity, **(C)** the SOD activity, **(D)** the T-AOC activity, **(E)** the MDA content. All data were expressed as the mean ± SD. **p < 0.01, *p < 0.05, and ns *p* > 0.05. SD was shown on the top of each column.

SOD, GSH-Px, CAT, T-AOC and MDA are precisely the key indicators for evaluating the antioxidant defense capacity and the degree of oxidative damage. Compared with the DSS group, preventive FA and Pdn supplementation significantly increased the activities of SOD, GSH-Px, CAT, T-AOC, and reduced the MDA content (*p* < 0.01, *p* < 0.05, Figures 3A–E). From the above results, preventive administration of FA and Pdn can alleviate the oxidative damage induced by DSS.

### 3.5 Colon histopathology in mice

**Figure 4:**
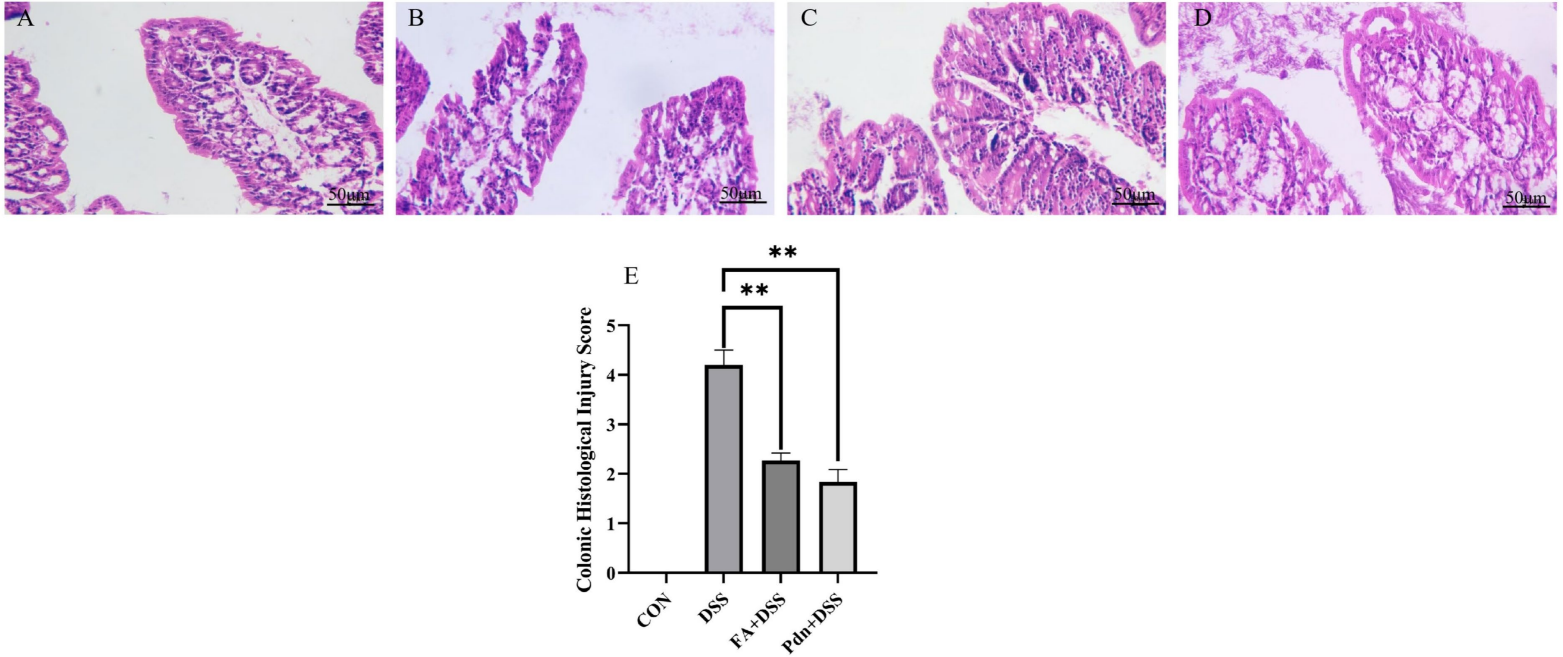
Histopathology of mice colon (*n* = 10 per group, 50 μm). The histopathology of mice colon was determined by HE staining method. **(A)** CON, **(B)** DSS group, **(C)** FA+DSS group, **(D)** Pdn+DSS group, **(E)** Colonic histological injury score. All data are expressed as the mean ± SD. **p < 0.01. SD was shown on the top of each column.

Colon histopathology with HE staining serves as the standard and ultimate evidence for evaluating the success of UC models. The extensive inflammation infiltration and crypt destruction were observed in the DSS group (Figures 4A,B). The colon injury was substantially attenuated in the FA+DSS group and Pdn+DSS groups (Figures 4C,D). The Colonic histological injury score was increased in DSS group, and it was lower in the FA+DSS group and Pdn+DSS group (Figure 4E) (*p* < 0.01) This study demonstrates that prophylactic administration of FA and Pdn can effectively alleviate the Colon histopathological damage in mice with DSS-induced colitis.

### 3.6 Gut microbiota diversity in mice

**Figure 5:**
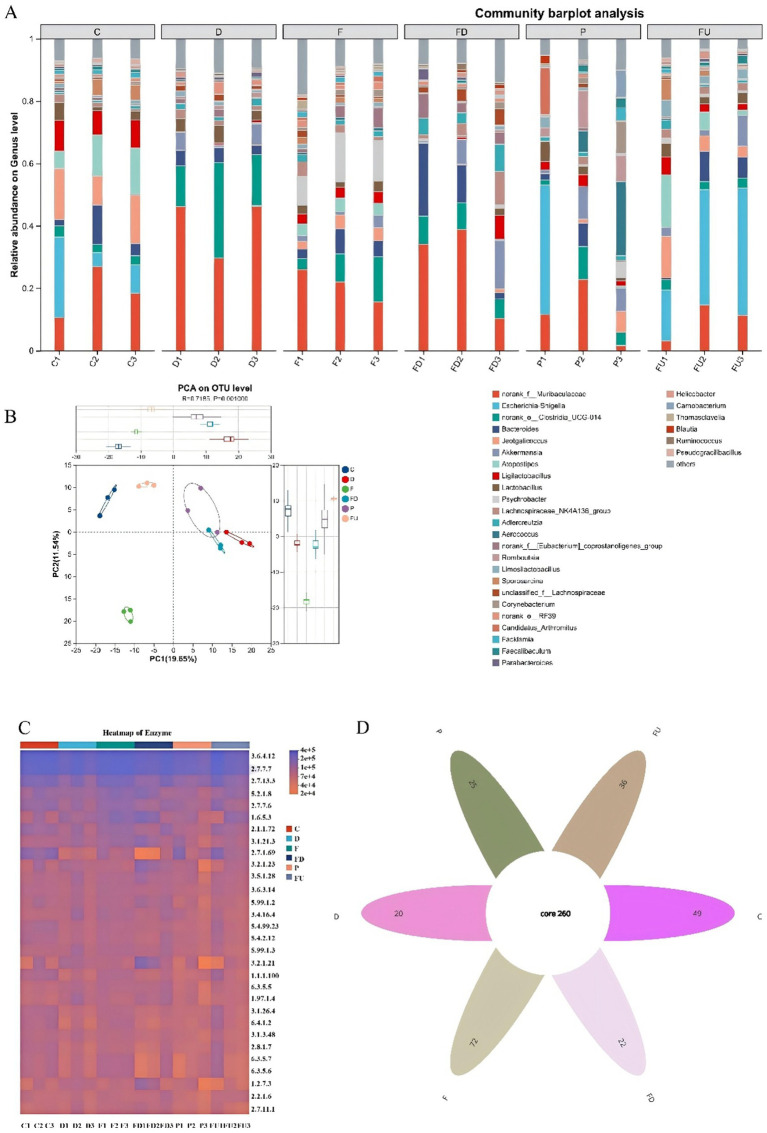
Gut microbiota diversity in mice (*n* = 3 per group). Gut microbiota composition was analyzed by sequencing of the 16S rRNA V4 gene region. C, CON, D, DSS group, FD, FA+DSS group, F, FA group, P, Pdn group, FU, Pdn+DSS group. **(A)** Community bar chart, **(B)** PCA graph, **(C)** Heatmap of Enzyme, **(D)** Venn diagram. C, CON, D, DSS group, FD, FA + DSS group, F, FA group, P, Pdn group, FU, Pdn+ DSS group.

The diversity of the intestinal microbiota using 16S rRNA sequencing method is of vital importance in UC. This disorder can directly trigger or exacerbate UC. *Muribaculaceae* and *Clostridia* was at a much higher level in the DSS group than that in the CON. A notable decrease was observed in the relative abundance of beneficial genera such as *Lactobacilli, Bifidobacteria, Bacteroides* in the DSS group. Following preventive administration of FA and Pdn, a restoration of the intestinal microbial community was observed, as evidenced by a significant increase of beneficial bacteria. Muribaculaceae and Clostridia were reduced, Bacteroides was increased in the FA+DSS and Pdn+DSS groups, indicating that FA and Pdn exerted a regulatory effect on gut microbiota composition. Based on these findings, it can be concluded that preventive administration of FA and Pdn improves the gut microbiota in mice (Figure 5).

### 3.7 The ultrastructural colon in mice

**Figure 6:**
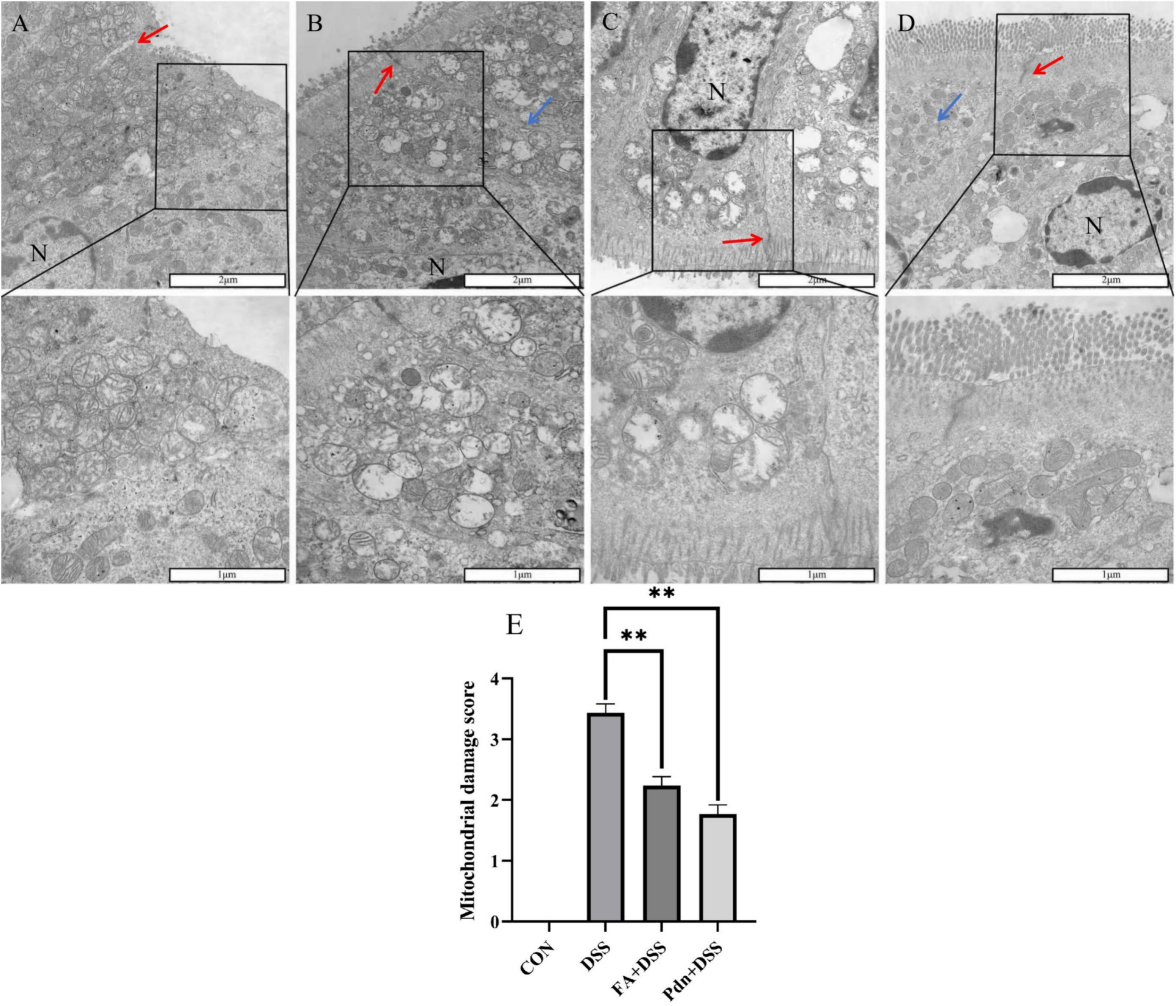
The ultrastructural changes of colitis (*n* = 10 per group, 2 μm). Ultrastructural changes in colon were examined with a transmission electron microscope. **(A)** CON, **(B)** DSS group, **(C)** FA+DSS group, **(D)** Pdn+DSS group, **(E)** mitochondrial damage score. The red arrow indicated intercellular junctions, and the blue arrow indicated the endoplasmic reticulum. N, the nucleus. All data are expressed as the mean ± SD. **p < 0.01, *p < 0.05, and ns *p* > 0.05. SD was shown on the top of each column.

The ultrastructural colon is key supplementary tools for revealing the damage of UC. As shown in Figure 6, In the DSS group, mitochondria were partially swollen with extravasated contents, and the marginalization of chromatin and endoplasmic reticulum dilation were observed. In the FA+DSS group and Pdn+DSS group, the lesion was alleviated. The mitochondria damage score was lower in the FA+DSS group and Pdn+DSS group compared the CON (*p* < 0.01). The Mitochondrial score was increased in DSS group, and mitochondria damage score was lower in the FA+DSS group and Pdn+DSS grou (*p* < 0.01) These findings indicate that FA and Pdn attenuate DSS-induced mitochondrial damage.

## 4 Discussion

In this study, preventive FA supplementation increased the body weight, the SOD, GSH-Px, CAT, T-AOC activity, IL-2 and IL-10 contents, reduced the IL-6, IL-1β, FC, FL, and the MDA content, NLRP3 protein expression in the DSS-induced UC mice. FA attenuated the extensive inflammation infiltration, crypt destruction and the mitochondrial damage in the DSS-induced UC mice. Meanwhile, FA can effectively improve the intestinal flora in mice. All test results consistently showed that, compared to the DSS group, both the FA and the Pdn prevention group exhibited significant and directionally similar improvements across all indicators, but there is no significance between these groups. FA has no side effects compared with Pdn. It indicates that preventive FA supplementation is helpful to alleviate the DSS-induced UC in mice.

DSS, a polyanionic derivative of dextran, was toxic to colonic epithelial cells and directly induced colon injury (Kiesler et al., 2015). DSS-induced colitis model has become one of the most widely UC mode (Xu et al., 2022; Eichele and Kharbanda, 2017). A concentration of 3% DSS can successfully establish the model within 5 to 10 consecutive days (Lu et al., 2022). In this experiment, DSS damaged colonic morphology, increased inflammatory cytokines, inhibited anti-inflammatory cytokines, antioxidant capacity, and disordered gut microbiota. It indicates that the UC mice model was successfully established. FA exerted anti-inflammatory effects through regulation of inflammatory cytokine levels and amelioration of oxidative stress (Liu et al., 2025). Huang et al. demonstrated that 80 mg/kg FA significantly inhibited the inflammation cytokines, enhanced intestinal antioxidant activity in the intestine (Huang et al., 2025). However, since the model-inducing drug used in this experiment was DSS acute modeling, the preventive intragastric administration days of 80 mg/kg were extended from 7 days to 10 days. Pdn was a synthetic glucocorticoid with anti-inflammatory, anti-allergic, and immunosuppressive properties (Bruscoli et al., 2021). A dose of 5 mg/kg prednisone was used as a positive drug control for the treatment of IBD (Mencarelli et al., 2016). This is the dosage for therapeutic use. In this experiment, Pdn was administered as a preventive drug, and the drug concentration was reduced to 4 mg/kg.

FC and FL are biomarkers for inflammation in UC (Chen et al., 2023). IL-2, IL-6 and IL-1β are pleiotropic pro-inflammatory cytokines and played a prominent role in the pathogenesis of IBD (Kaur et al., 2020; Mao et al., 2018). IL-10 is a well-defined anti-inflammatory cytokine (Li et al., 2024). These cytokines performed multiple functions at sites of inflammation (Li and Zhang, 2023). In this experiment, FA markedly reduced the elevation of FC, FL, IL-1β and IL-6, and promoted the increase of IL-2 and IL-10. Fecal Cp and Lf levels had almost equivalent abilities in detecting clinical remission and tended to be higher in UC patients (Yamakawa et al., 2024). The reduction of the FC and FL in the FA+DSS and Pdn+DSS group indicates that the inflammation of UC was alleviated. IL-1β induced the expression of proinflammatory cytokine, promoting the amplification of inflammatory response (Pirklbauer et al., 2021). The deregulation of IL-6 was associated with chronic inflammation (Uciechowski and Dempke, 2020). The reduction of the IL-1β and IL-6 in the FA+DSS and Pdn+DSS group indicates that the inflammation of UC was alleviated. The function of IL-10 is to inhibit the production of pro-inflammatory cytokines, suppressing the inflammatory response (Li et al., 2024). The increase of IL-10 in the FA+DSS and Pdn+DSS group indicates that the anti-inflammation activity of UC mice is promoted. It indicates that the inflammation of UC was attenuated. However, in this experiment, the inflammation cytokine, IL-2, was increased. The both pro-inflammatory and anti-inflammatory function helps IL-2 to regulate the immune balance (Chen et al., 2024; Phillips et al., 2020). The promotion of IL-2 is similar to Linghu’s experiment (Linghu et al., 2022). Considering the pivotal role of the NLRP3 inflammasome in inflammatory responses, these results implied that FA might exert its anti-inflammatory effects. This study provides evidence that FA attenuates NLRP3-mediated inflammation. Ekhtiar et al. (2025) verified that FA reduced the expression of inflammatory factors, which provided theoretical basis for this experiment. FA attenuated the extensive inflammation infiltration, crypt destruction and the mitochondrial damage in the DSS-induced UC mice. The recover of the colitis morphology contributes to the inhibition of inflammation cytokines. Pdn elicited anti-inflammatory and immunosuppressive effects via pleiotropic modulation of immune function (Babbar et al., 2021). In this experiment, FA has the same protective effect on the inflammation with Pdn, but there is no significant difference between the two groups. Pdn has side effect in the treatment of UC. It indicates that FA inhibited the inflammation cytokine and promoted the anti-inflammation cytokines.

Natural antioxidant compounds exhibit ROS scavenging and increase antioxidant defense capacity to inhibit pro-oxidative enzymes, which may be useful in IBD treatment (Sahoo et al., 2023). In this experiment, the elevation of SOD, GSH-Px, and CAT activity induced by FA and Pdn indicated that FA restored the anti-oxidative function of UC mice. Another evidence showed that FA attenuated oxidative stress-induced cardiomyocyte injury (Sun et al., 2021). It supports our findings of FA on the antioxidative activity in the UC. Reactive oxygen species (ROS) and reactive nitrogen species (RNS), are produced at abnormally high levels in UC. Their destructive effects may contribute to the disease’s initiation and propagation. MDA can be measured in the blood and stool of patients with IBD (Muro et al., 2024). The reduction of MDA further confirmed FA and Pdn’s ability to mitigate oxidative damage in UC mice. T-AOC was a comprehensive indicator reflecting the overall antioxidant capacity (Xie et al., 2025). In this experiment, FA and Pdn restored T-AOC activity in UC mice. FA attenuated extensive inflammation infiltration, crypt destruction and mitochondria damage in colon of this experiment. The recover of the extensive inflammation infiltration, crypt destruction and the mitochondrial damage in the colitis morphology contributes to the increase of antioxidative function. In the gastrointestinal tract, infections enhanced ROS generation through pro-inflammatory cytokine upregulation (Sahoo et al., 2023). In this experiment, the reduction of the pro-inflammatory cytokine and the anti- inflammatory cytokine contribute to the inhibition of the oxidative stress in UC mice. It indicates that FA and Pdn promotes the antioxidative function of UC mice.

UC diminished intestinal microbiota diversity, depleting beneficial and expanding pathogenic bacteria (Yu Z. et al., 2023). DSS administration disrupted the gut microbiota, precipitating dysbiosis (Xu et al., 2021). In this experiment, the reduction of the pro-inflammatory cytokine also contributes to the recovery of the gut microbiota. Similarly, Clostridia, a component of the gut microbiota, is often associated with gut health in many studies. It was observed that the FA+DSS and Pdn+DSS group exhibited a significant reduction in bacteria of the class Clostridia. Gut microbiota dysbiosis triggered inflammation (Clinton and Cross, 2023). This increase promotes its ability to suppress inflammation.

Certain species within the genus *Clostridium* were important intestinal pathogens, and the reduction in their colonies could directly attenuate enteritis. Bacteroides, as one of the core members of the gut microbiota, played a crucial role in suppressing UC. Its mechanisms of action involve maintenance of microbial ecological balance, and anti-inflammatory effects mediated by metabolites. DSS-induced microbial disorder contributes to intestinal inflammation (Yang and Merlin, 2024). In the FA+DSS and Pdn+DSS group, a notable increase in *Bacteroides* was detected. *Bacillus*, also known as the spore-forming genus, and demonstrated multifaceted beneficial effects in suppressing IBD. Mechanistically, it acted by modulating gut microbiot. FA significantly changed gut microbiota composition in zebrafish (Yin et al., 2022). In the FA+DSS group of mice, a significant increase in the abundance of *bacillus* was observed. These findings collectively indicated that FA modulated the gut microbiota. *Muribaculaceae* plays essential roles in maintaining intestinal barrier integrity, modulating immune responses, and regulating energy metabolis, and was significantly depleted in the FA+DSS and Pdn+DSS group, suggesting that FA exposure under DSS-induced colitis may perturb microbial community homeostasis and contribute to dysbiosis. Conversely, a notable increase was observed in the relative abundance of beneficial genera such as *Lactobacilli* and *Bifidobacteria*. It indicates that they may be closely associated with the recovery pathological process in the FA+DSS and Pdn+DSS group. Additionally, FA increases the *Bacteroides* population, decreased dysbiosis and reduce multiple harmful consequences. A significant increase in *Bacteroides* was indeed observed in the FA+DSS and Pdn+DSS group. FA attenuated extensive inflammation infiltration, crypt destruction and mitochondria damage in colon of this experiment. Therefore, it can be concluded that FA can effectively improve the intestinal flora disorder in mice.

Therefore, FA and Pdn attenuate the UC through recover of the colon histopathology, reduction of inflammation, promotion of antioxidative function, regulation of intestinal flora. This provides a solid experimental foundation for its development as a novel and safe preventive agent for UC. The difference between the FA and Pdn group will be conducted in the future.

## Data Availability

The data presented in the study are deposited in the the NCBI SRA repository, accession number PRJNA1445482.
