# Supplementary figures and images for: Preventive effect of ferulic acid on dextran sulfate sodium-induced ulcerative colitis in mice
Source: Front Microbiol. 2026 Apr 13;17:1767221. doi: 10.3389/fmicb.2026.1767221 (PMC13111449; doi:10.3389/fmicb.2026.1767221)

Beta Actin-1: 42 KDa

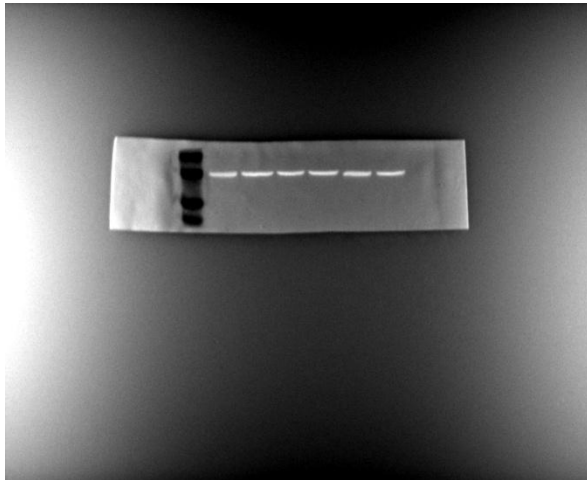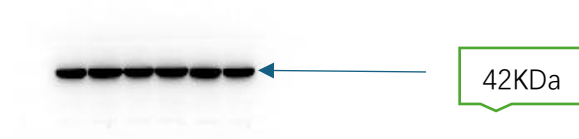

Beta Actin-2

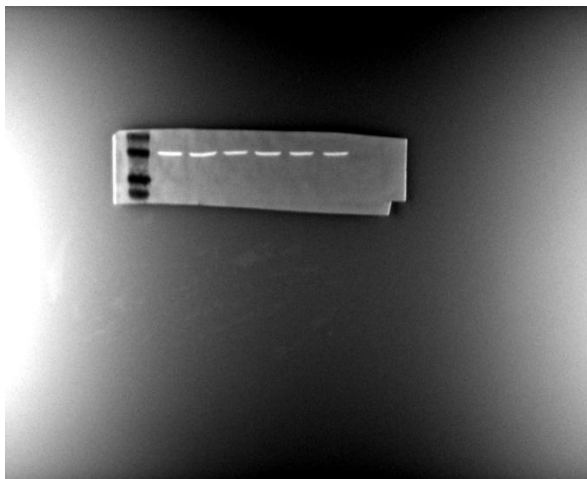

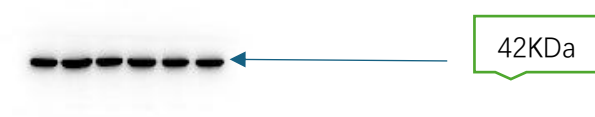

Beta Actin-3

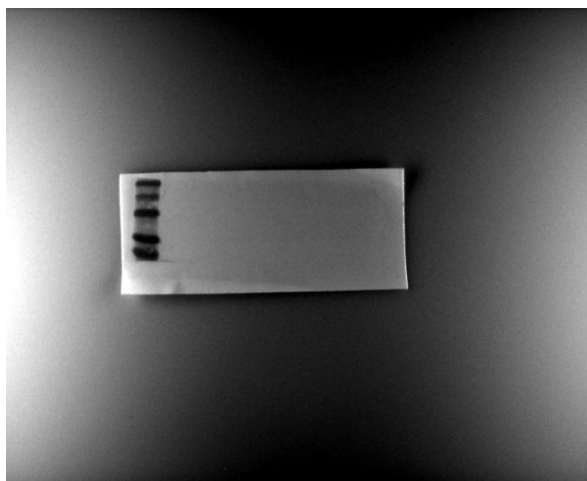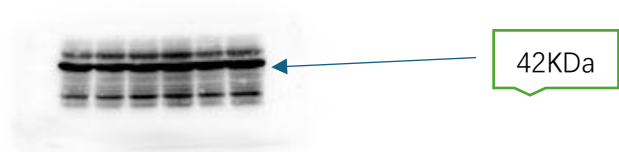

NLRP3-1: 110KDa

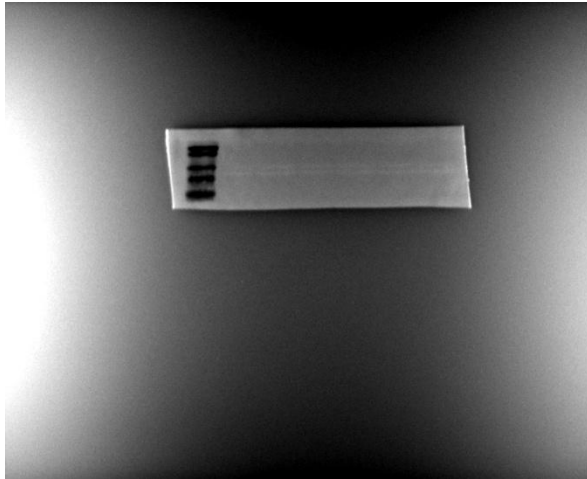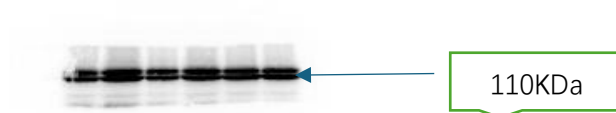

NLRP3-2

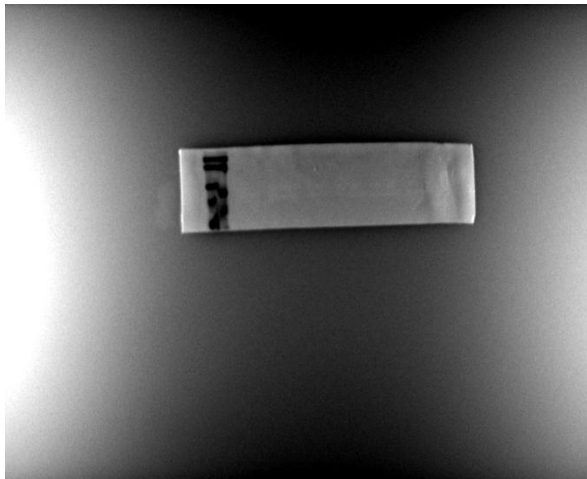

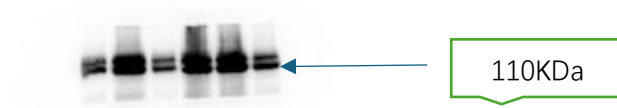

NLRP3-3

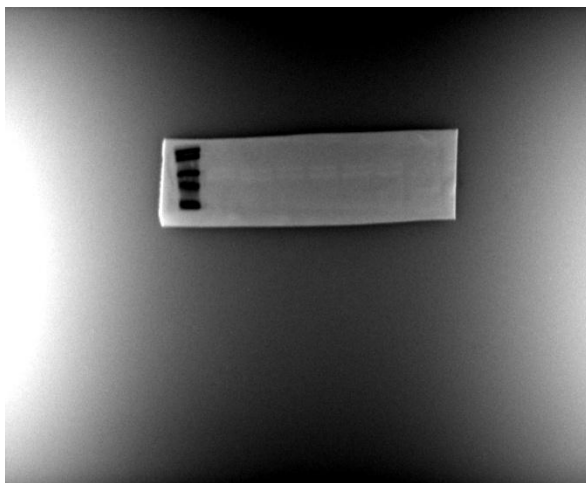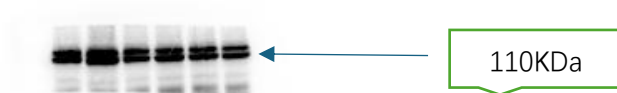

Supplement: Supplementary file 1 [file Data_sheet_1.pdf]

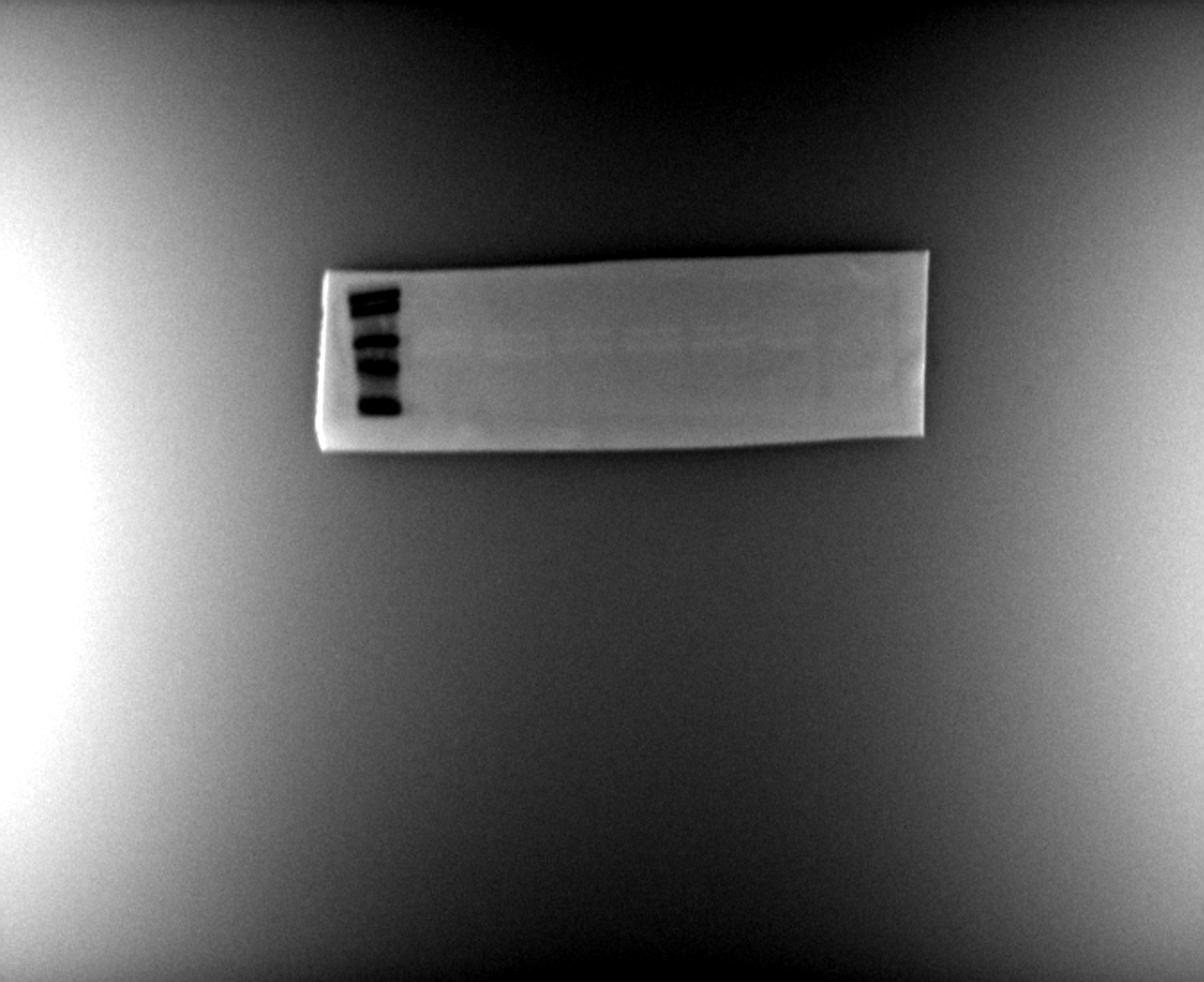

Supplement: Supplementary file 2 [file Data_sheet_2.zip › Figure 2M WB images/NLRP3/NLRP3 1 in Fig 2M PVDF membrane.jpg]

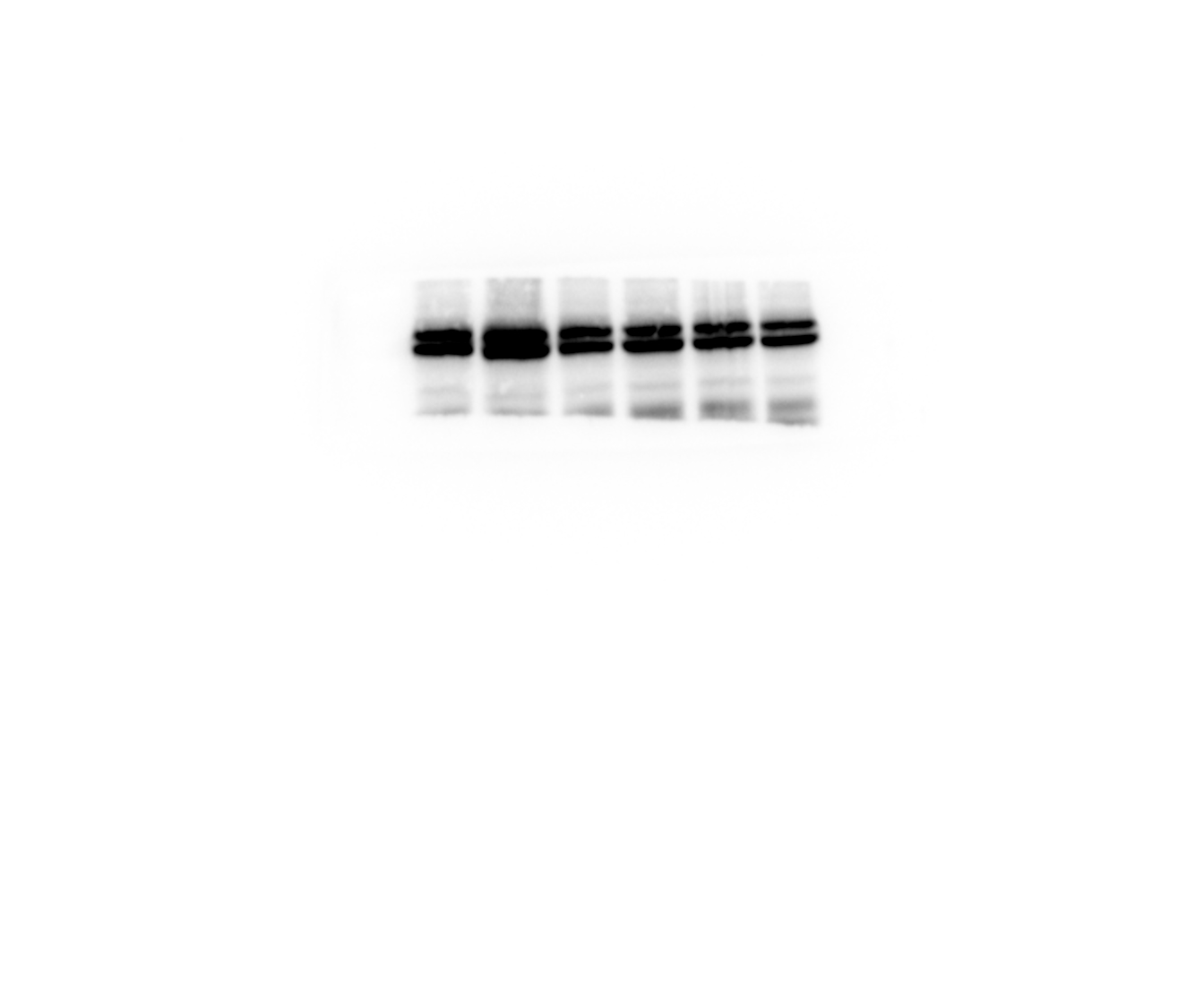

Supplement: Supplementary file 2 [file Data_sheet_2.zip › Figure 2M WB images/NLRP3/NLRP3 1 in Fig 2M.jpg]

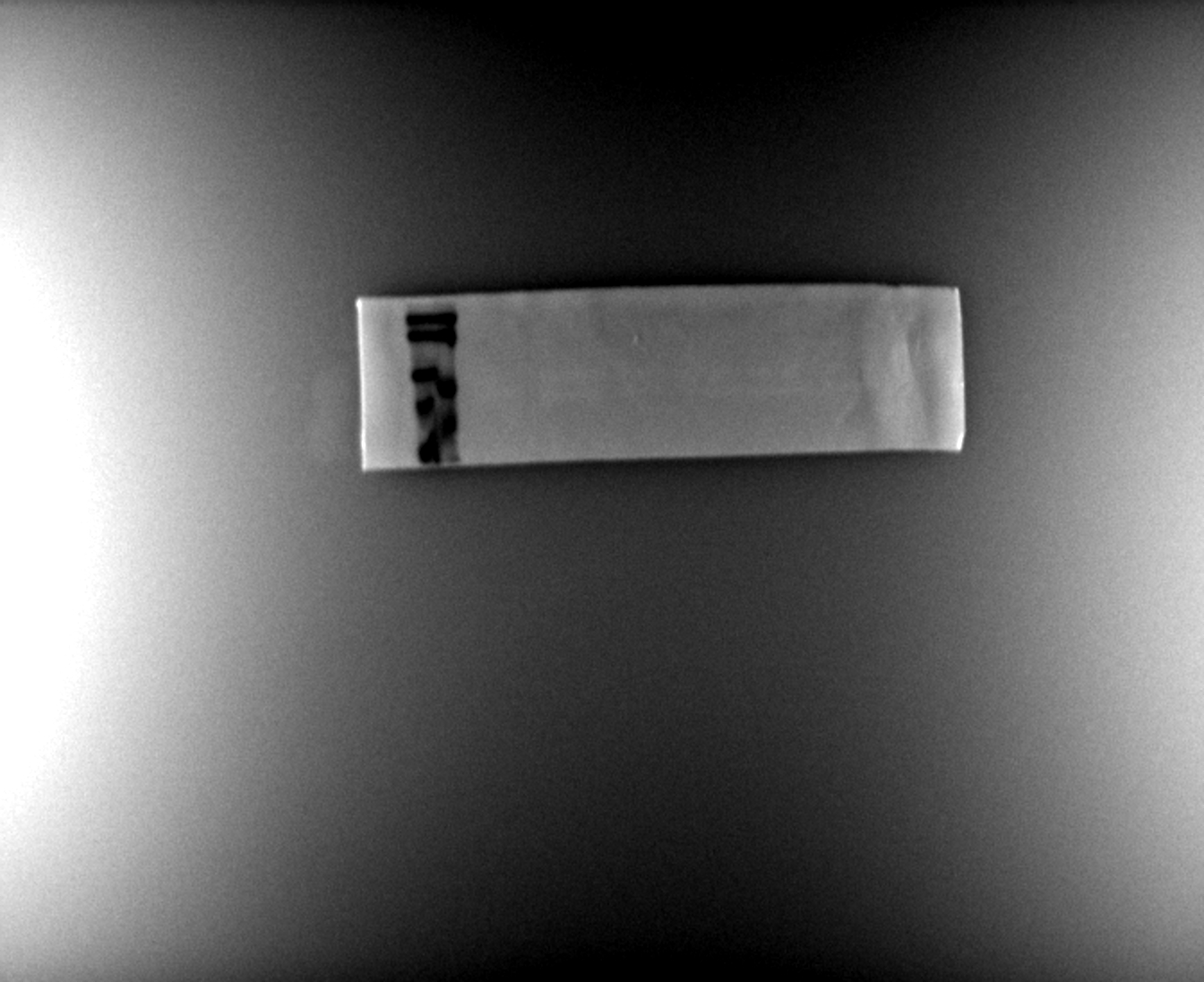

Supplement: Supplementary file 2 [file Data_sheet_2.zip › Figure 2M WB images/NLRP3/NLRP3 2 in Fig 2M PVDF membrane.jpg]

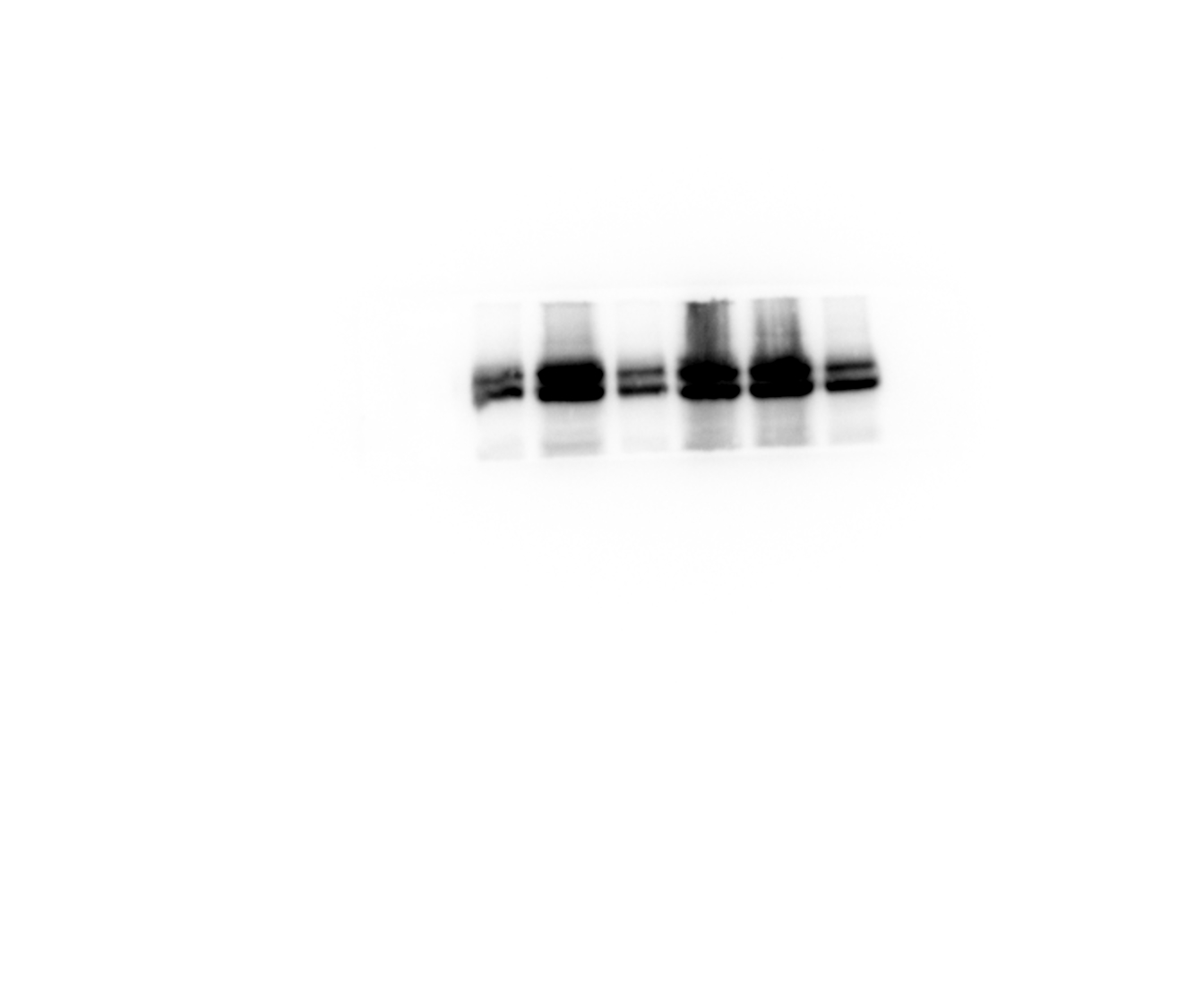

Supplement: Supplementary file 2 [file Data_sheet_2.zip › Figure 2M WB images/NLRP3/NLRP3 2 in Fig 2M.jpg]

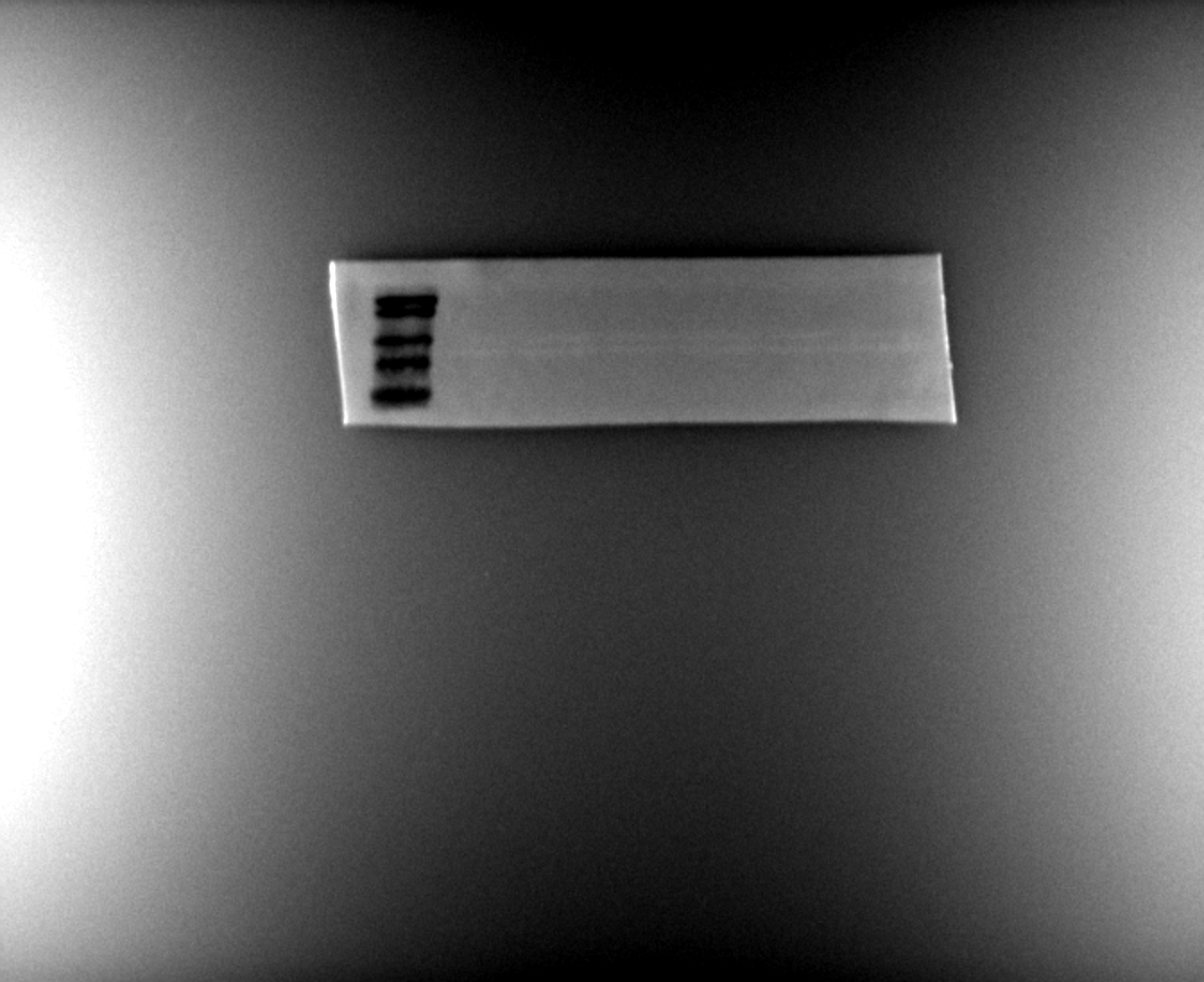

Supplement: Supplementary file 2 [file Data_sheet_2.zip › Figure 2M WB images/NLRP3/NLRP3 3 in Fig 2M PVDF membrane.jpg]

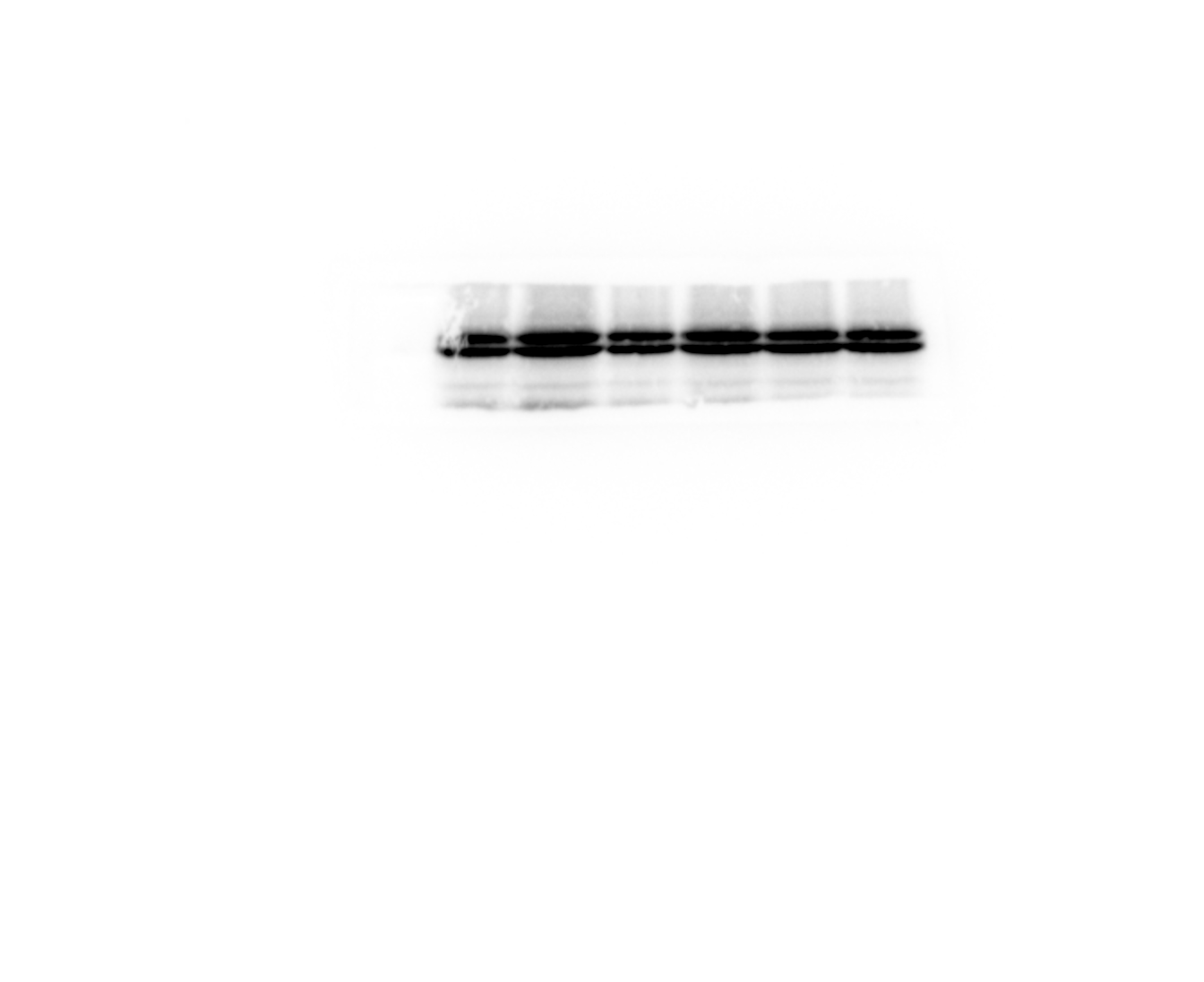

Supplement: Supplementary file 2 [file Data_sheet_2.zip › Figure 2M WB images/NLRP3/NLRP3 3 in Fig 2M.jpg]

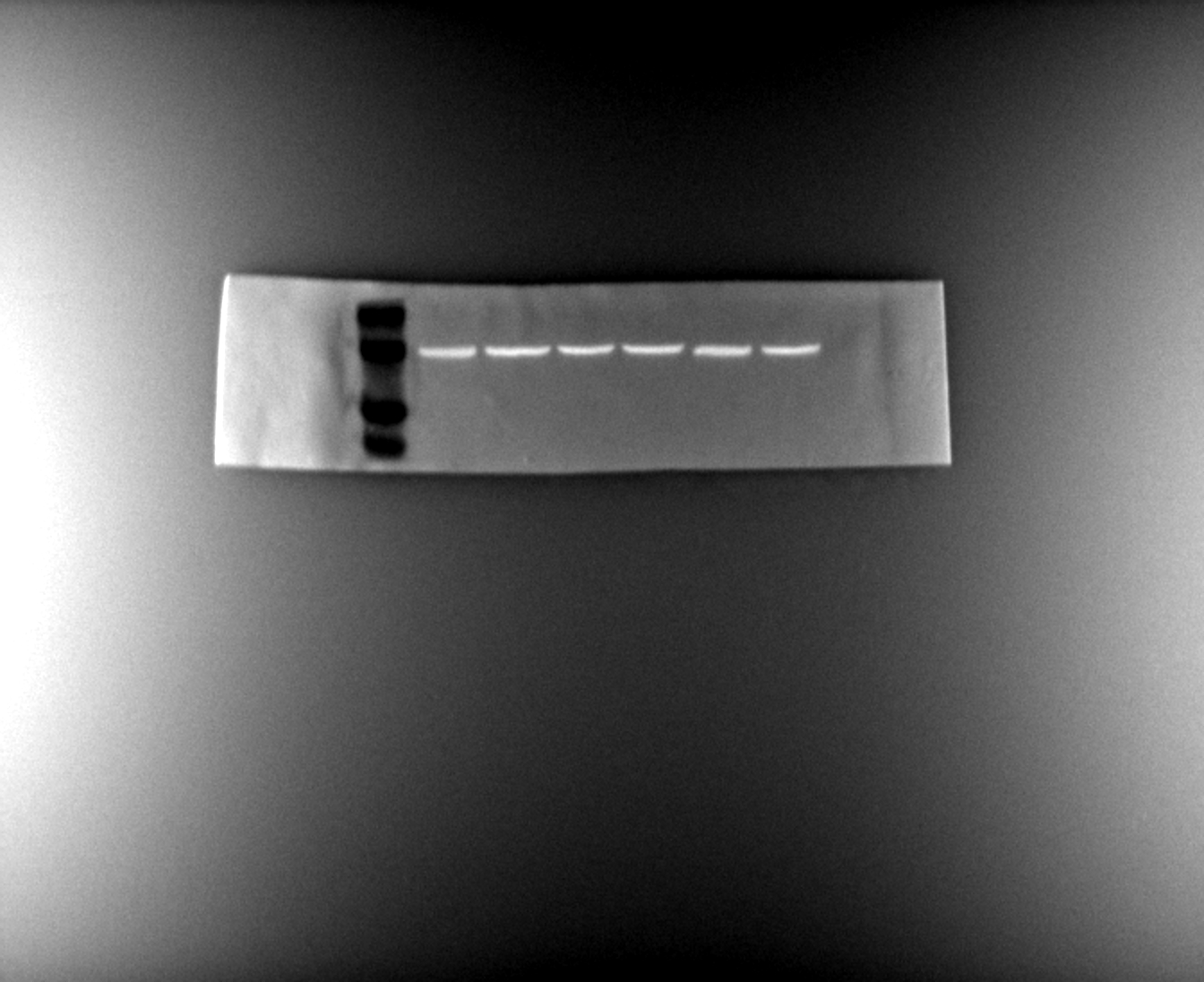

Supplement: Supplementary file 2 [file Data_sheet_2.zip › Figure 2M WB images/β-actin/β-actin 1 in Fig 2M PVDF membrane.jpg]

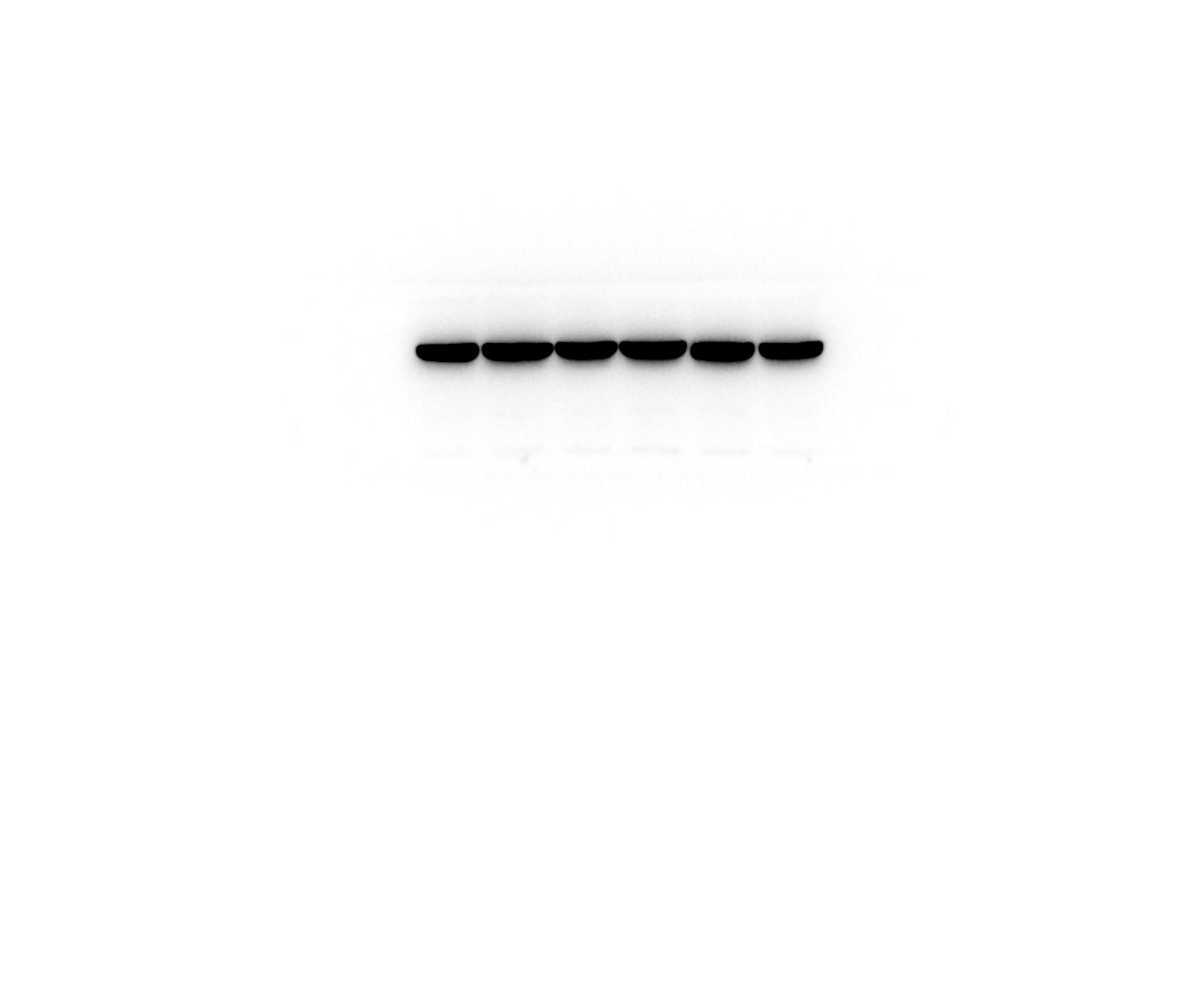

Supplement: Supplementary file 2 [file Data_sheet_2.zip › Figure 2M WB images/β-actin/β-actin 1 in Fig 2M.jpg]

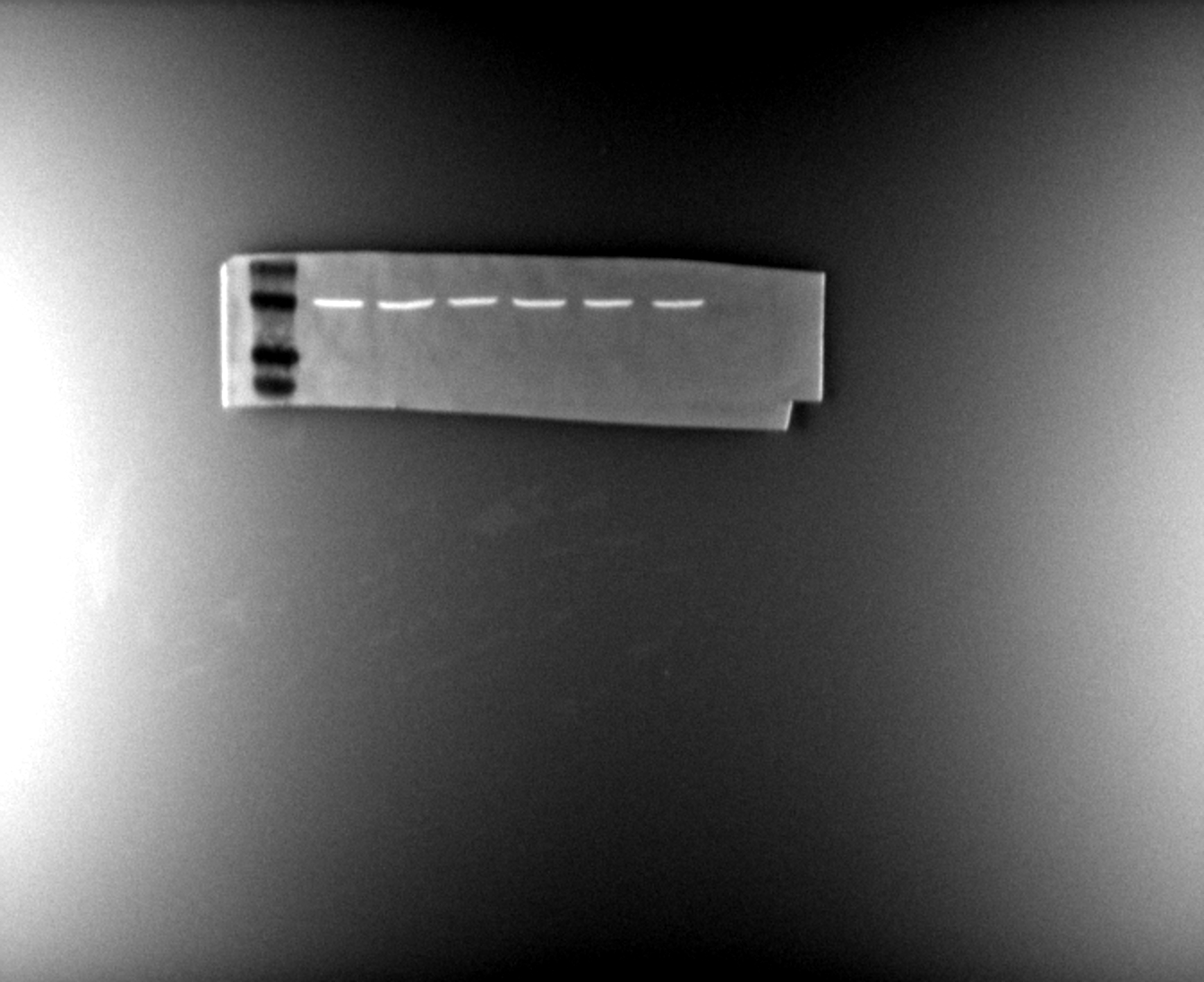

Supplement: Supplementary file 2 [file Data_sheet_2.zip › Figure 2M WB images/β-actin/β-actin 2 in Fig 2M PVDF membrane.jpg]

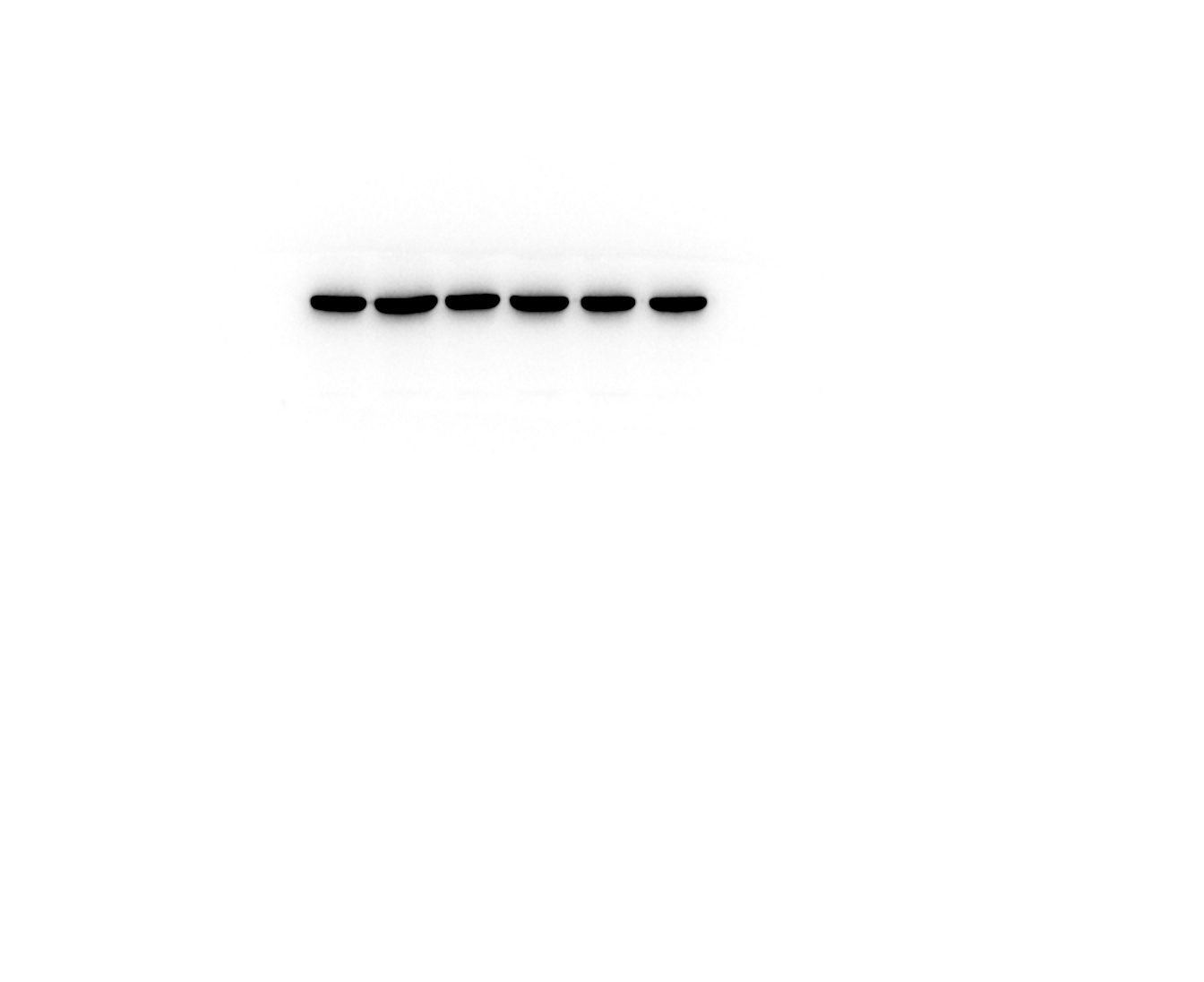

Supplement: Supplementary file 2 [file Data_sheet_2.zip › Figure 2M WB images/β-actin/β-actin 2 in Fig 2M.jpg]

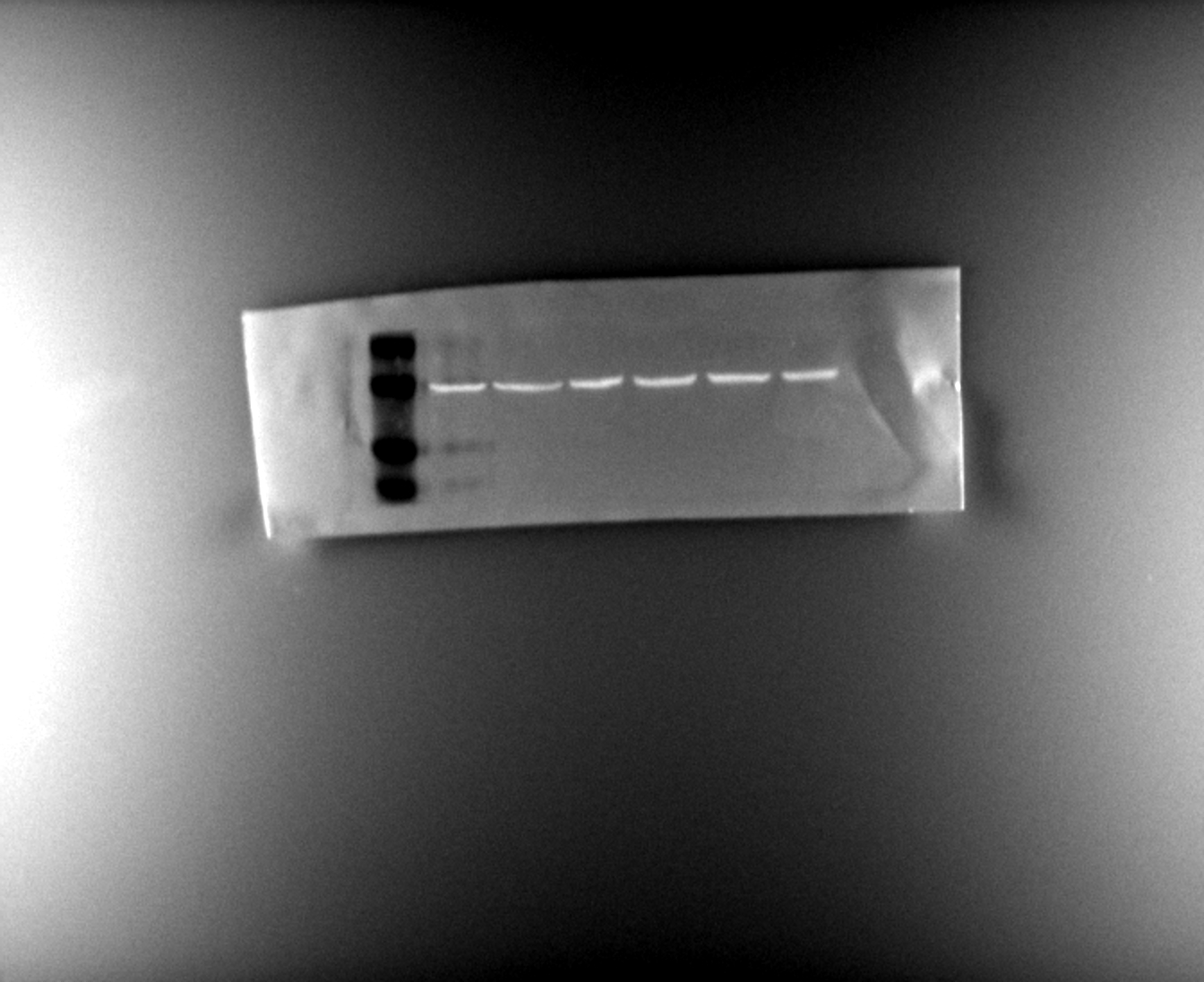

Supplement: Supplementary file 2 [file Data_sheet_2.zip › Figure 2M WB images/β-actin/β-actin 3 in Fig 2M PVDF membrane.jpg]

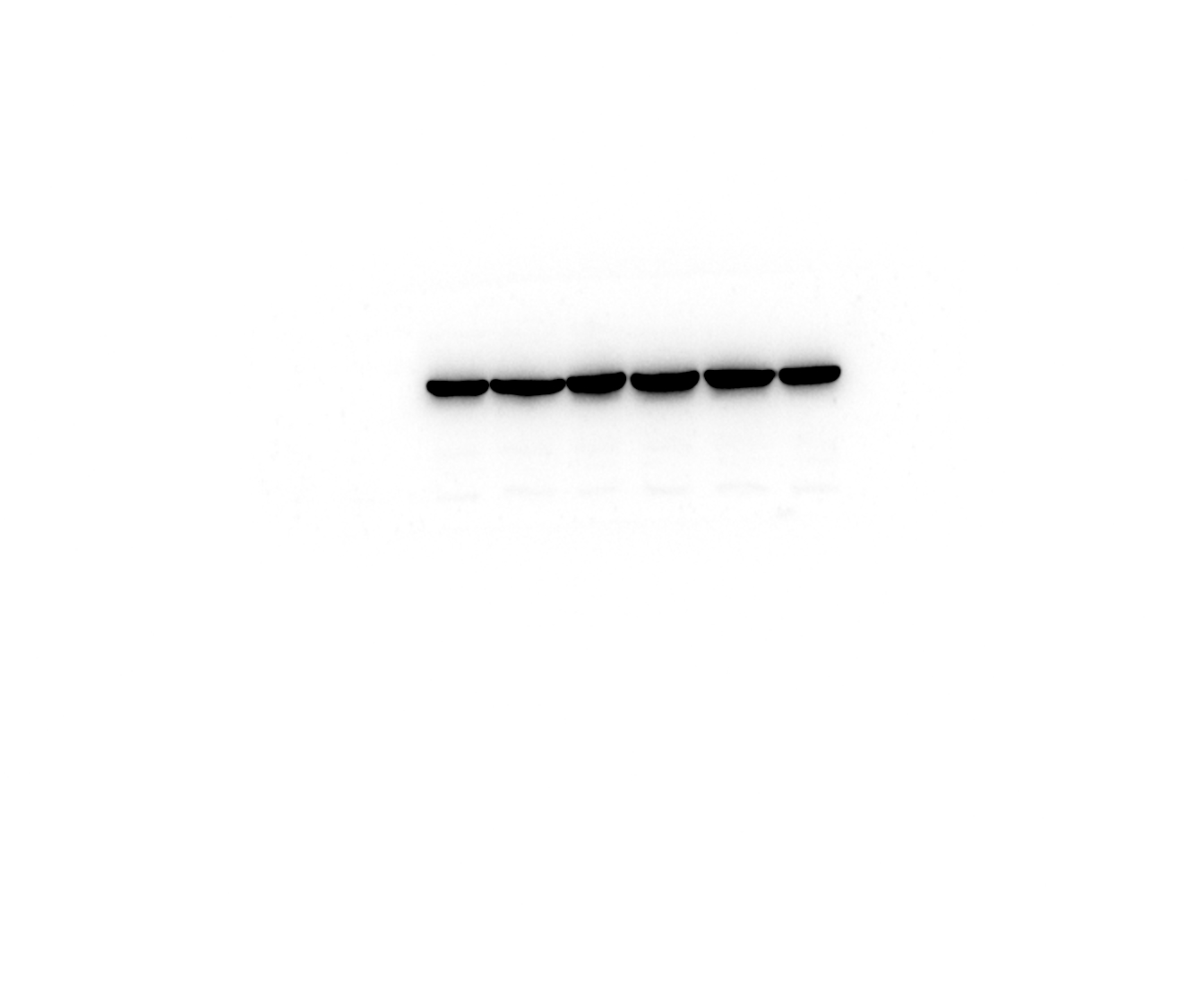

Supplement: Supplementary file 2 [file Data_sheet_2.zip › Figure 2M WB images/β-actin/β-actin 3 in Fig 2M.jpg]
